# Editorial: The Neural Basis of Human Prosocial Behavior

**DOI:** 10.3389/fpsyg.2019.02058

**Published:** 2019-09-10

**Authors:** Yefeng Chen, Hang Ye, Chao Liu, Qi Li

**Affiliations:** ^1^College of Economics and Interdisciplinary Center for Social Sciences, Zhejiang University, Hangzhou, China; ^2^Center for Economic Behavior and Decision-Making, School of Economics, Zhejiang University of Finance and Economics, Hangzhou, China; ^3^State Key Laboratory of Cognitive Neuroscience and Learning & IDG, McGovern Institute for Brain Research, Beijing Normal University, Beijing, China; ^4^Key Laboratory of Behavioral Science, Institute of Psychology, Chinese Academy of Sciences, Beijing, China

**Keywords:** neural basis, prosocial behavior, neural mechanisms, neural imaging, brain stimulation

With the rise of laboratory and field experimental economics, the famous prisoner's dilemma, public good, dictator, ultimatum, and trust games have become the classical paradigms of studying prosocial behavior (Güth et al., 1982; Berg et al., 1995; Fehr and Gächter, 2002; Camerer, 2003). Due to the increasing use of functional magnetic resonance imaging (fMRI), transcranial magnetic stimulation (TMS) and transcranial direct current stimulation (tDCS) with human subjects playing economic games, the neural basis of prosocial behavior has been uncovered by a large amount of neural imaging and stimulating research (Rilling et al., 2002; Sanfey et al., 2003; de Quervain et al., 2004; Knoch et al., 2006; Krueger et al., 2007). A wide range of brain areas including, but not limited to the prefrontal cortex, orbitofrontal cortex, cingulate cortex, striatum, and amygdale have been revealed highly correlated or causally related with prosocial behaviors.

A number of hypotheses such as empathy, altruism, reciprocity, inequality aversion, or guilt aversion preferences have been considered as motives promoting prosocial behavior. However, the neural bases of these different preferences have seldom been revealed and the mechanisms of how these preferences influence prosocial behavior have rarely been discussed. Moreover, since prosocial behavior may be due to the cooperative work of several brain areas (neural network), it is essential to integrate findings from difference disciplines including psychology, economics, neuroscience, and to nearly all the social and behavioral sciences.

The present Research Topic of Frontiers in Psychology aims to bring a collection of research revealing the neural basis of human prosocial behavior. Totally 14 articles composing this unique Frontiers Research Topic in different types of prosocial behavior.

There are 3 review articles included in this volume. Luo summarize the research on the neural basis of different types of pro-social behaviors and describe a common shared neural circuitry of these pro-social behaviors. This review introduces several widely used approaches to develop new insights into understanding prosocial behaviors by combining the game theory of economics with neuroscience technologies. Zheng et al. summarize models of the emotional influence on fairness-related decision making and the corresponding behavioral and neural evidence. In their view, the future research on fairness-related decision making should focus on inducing incidental social emotion, avoiding irrelevant emotion when regulating, exploring the individual differences in emotional dispositions, and strengthening the ecological validity of the paradigm. Liu et al. review neuroimaging studies on social networks, and probe into the connection between individuals' social network size and neural mechanisms. They find there are two main methods to measure the social network size. One is Social Network Index and the other is Social Network Questionnaires. These two measurements in view of the hierarchical organization of social networks are carefully examined in this paper. And the authors reveal that the two assessments are dissimilar in effect. This finding sheds new light on the understanding of the subtle distinctions among various social network assessments.

Adopting givesome games and public good dilemma, Liu et al. explored social interaction patterns between the disabled and abled people. This is the only one behavioral study but not neural study in this volume. However, this study is quite interesting using a special sample. They found disabled people were more likely to interact with the disabled people, while the abled people preferred to interact with the abled people; comparing with the abled people, the disabled people had higher cooperation; they also revealed that advantage in the number of the disabled people could reverse their disadvantage in the identity. The results provide related theoretical support for the disabled people's federation and communities when carrying out activities for the disabled people.

All the remaining 10 papers explore the neural basis of different types of prosocial behavior using neuroimaging and brain stimulation approaches such as fMRI, TMS, tDCS, ERP, and so on.

Using the event-related potential (ERP) technique, Liu et al. explored neural mechanisms underlying the processing of evaluating altruistic outcomes when self-interests are sacrificed. Their ERP results showed that when evaluating another person's outcomes in the low-empathy condition, an inversed FRN effect occurred. But this kind of effect did not appear in the high-empathy condition. This study suggest that empathy could modulate the neural responses to altruistic outcomes in which increasing welfare of others could result in a cost of the self.

On the topic of fairness and inequity aversion, Li et al. provided behavioral and electrophysiological data to demonstrate that advantageous inequity aversion may differ as a function of the individual's role in determining allocations. If the individual cannot decide to distribute, this kind of inequity aversion will disappear. In their functional MRI study, Wei et al. investigated how social support affects the responders' fairness considerations and related decision-making processes in the ultimatum game. They demonstrated that the fairness-related decision-making processes are context-dependent and are modulated by social support.

By manipulating prestige-based social status, Blue et al. found that participants who played the role of investors in TG tended to be more affected by higher status Trustee promises than by lower status Trustee promises, despite the equal reinforcement schedule across conditions. Their findings suggest that honesty perception is affected by social status at both a behavioral and neural level, and that subjective socio-economic status may modulate this effect.

In the research on cooperation and punishment, using a linear asymmetric PG, Li et al. demonstrated the effect of the rLPFC on a priori normative beliefs without threats of external punishment through tDCS. Their finding reveals that rLPFC stimulation affects beliefs in the cooperation norm. As the author said, this research is a promising step toward understanding how neurobiological mechanisms are connected to beliefs in cooperation norms. In another study, for the first time, Li et al. compared the different neural processes of fourth-party evaluation on third-party help and punishment. Their ERP results revealed that fourth-party bystanders' FRN amplitudes were modulated by the third-party behaviors.

Regarding the study of deception, Gao et al. investigated the effect of modulating the activity of the DLPFC on deception. They conducted a between-subject design in a signaling framework of deception. Their results demonstrated the important role of DLPFC in modulating self-interested driven deceptive behavior. And they also found that in the sham stimulation treatment, males were more honest than females, while such gender difference disappeared in the right anodal/left cathodal stimulation treatment. Moreover, Tang et al. is the first study to investigate how activity in rTPJ affects deception in fairness related moral hypocrisy. They used a revised version of dictator game to examine the role of self-centered and other-regarding concerns in deception through stimulating rTPJ by tDCS. They found that deception in moral hypocrisy was increased by revealing appearing fair without true fairness to recipients than not. And this effect was decreased by anodal stimulation on rTPJ rather than cathodal and sham stimulation.

Finally, there are 2 paper focus on the moral judgment. In Ying et al.'s functional magnetic resonance imaging study, the participants evaluated the degree of disgust using sentences related to mild moral violations with different types of behavioral agents including the mother and stranger. They doubly dissociated two insular components in the processing of moral transgression events, and found that in the stranger condition, the component located in the posterior region was more activated. While in the case of mother condition, the other component located in the anterior region was more activated. This study provided key evidence for understanding the principle of embodied cognition. In addition, they also demonstrated that high-level moral disgust is built on more basic disgust via a mental construction approach through a process of embodied schemata. Using tDCS which allows cortical excitability to be directly manipulated, Zheng et al. investigated whether modulating the excitability of the bilateral DLPFC (or TPJ) can directly influence participants' moral judgments by affecting their cognitive reasoning or emotional processes. They observed that activating the right DLPFC as well as inhibiting the left DLPFC led to less utilitarian judgments especially in moral-personal conditions, indicating that the right DLPFC plays an crucial role in moral judgments. Their findings provide important information regarding the impact of tDCS on the DLPFC of healthy participants, especially with respect to moral-personal dilemmas.

Overall, we believe that the research presented in this topic can promote a better understanding of neural basis of prosocial behavior.

## Author Contributions

All authors listed have made a substantial, direct and intellectual contribution to the work, and approved it for publication.

### Conflict of Interest Statement

The authors declare that the research was conducted in the absence of any commercial or financial relationships that could be construed as a potential conflict of interest.
